# Relationship between time from symptom’s onset to diagnosis and prognosis in patients with symptomatic colorectal cancer

**DOI:** 10.1186/s12885-022-09990-7

**Published:** 2022-08-22

**Authors:** Magdalena Esteva, Alfonso Leiva, María Ramos-Monserrat, Alejandro Espí, Luis González-Luján, Francesc Macià, Cristiane Murta-Nascimento, María A. Sánchez-Calavera, Rosa Magallón, Vanesa Balboa-Barreiro, Teresa Seoane-Pillado, Sonia Pertega-Díaz

**Affiliations:** 1 Department of Primary Care, Primary Care Research Unit, Majorca, Baleares Health Service [IbSalut]. Escola Graduada 3, 07001 Palma, Spain; 2 Balearic Islands Health Research Institute (IdISBa), University Hospital Son Espases, Edificio S, Carretera de Valldemossa, 79, 07120 Palma, Majorca, Spain; 3 Preventive Activities and Health Promotion Research Network (REDIAPP), Barcelona, Spain; 4 Research Network On Chronicity, Primary Care, and Health Promotion (RICAPPS) , Madrid, Spain; 5 University of the Balearic Islands (UIB), Carretera de Valldemossa, km 7.5, 07122 Palma, Spain; 6 Balearic Islands Public Health Department, C/ Jesus 38A, 07010 Palma, Spain; 7 Department of Surgery, University of Valencia, Avenida Blasco Ibáñez 15, 46010 Valencia, Spain; 8 Serrería II Primary Care Centre, Valencia Institute of Health, Pedro de Valencia 26, 46022 Valencia, Spain; 9 Epidemiology and Evaluation Unit, Hospital del Mar, Passeig Marítim 25-29, 08003 Barcelona, Spain; 10 Department of Medicine, University of Zaragoza, Building A, 50009 Saragossa, Spain; 11 Las Fuentes Norte Health Center, Calle Dr. Iranzo 69, 50002 Saragossa, Spain; 12 Instituto de Investigación Sanitaria Aragón (IIS Aragón), Saragossa, Spain; 13 Centro de Salud Arrabal, Andador Aragüés del Puerto, 3, 50015 Saragossa, Spain; 14 Nursing and Healthcare Research Group, Rheumatology and Health Research Group, Instituto de Investigación Biomédica de A Coruña (INIBIC), Complexo Hospitalario Universitario de A Coruña (CHUAC), Sergas. Universidade da Coruña (UDC), As Xubias, 15006. A Coruña, Spain

**Keywords:** Colorectal neoplasm, Prognosis, Delayed diagnosis, Time factors, Cancer-specific mortality

## Abstract

**Background:**

Controversy exists regarding the relationship of the outcome of patients with colorectal cancer (CRC) with the time from symptom onset to diagnosis. The aim of this study is to investigate this association, with the assumption that this relationship was nonlinear and with adjustment for multiple confounders, such as tumor grade, symptoms, or admission to an emergency department.

**Methods:**

This multicenter study with prospective follow-up was performed in five regions of Spain from 2010 to 2012. Symptomatic cases of incident CRC from a previous study were examined. At the time of diagnosis, each patient was interviewed, and the associated hospital and clinical records were reviewed. During follow-up, the clinical records were reviewed again to assess survival. Cox survival analysis with a restricted cubic spline was used to model overall and CRC-specific survival, with adjustment for variables related to the patient, health service, and tumor.

**Results:**

A total of 795 patients had symptomatic CRC and 769 of them had complete data on diagnostic delay and survival. Univariate analysis indicated a lower HR for death in patients who had diagnostic intervals less than 4.2 months. However, after adjustment for variables related to the patient, tumor, and utilized health service, there was no relationship of the diagnostic delay with survival of patients with colon and rectal cancer, colon cancer alone, or rectal cancer alone. Cubic spline analysis indicated an inverse association of the diagnostic delay with 5-year survival. However, this association was not statistically significant.

**Conclusions:**

Our results indicated that the duration of diagnostic delay had no significant effect on the outcome of patients with CRC. We suggest that the most important determinant of the duration of diagnostic delay is the biological profile of the tumor. However, it remains the responsibility of community health centers and authorities to minimize diagnostic delays in patients with CRC and to implement initiatives that improve early diagnosis and provide better outcomes.

**Supplementary Information:**

The online version contains supplementary material available at 10.1186/s12885-022-09990-7.

## Introduction

Colorectal cancer (CRC) is one of the most common cancers and a leading cause of cancer deaths in Europe [1]. Although the 5-year overall survival rate in Europe has improved during recent decades, these survival rates are still only about 60%, indicating a need for improvement [2]. The prognosis of a patient with CRC depends on the stage at diagnosis, and many health organizations have therefore recently focused on early diagnosis patients with symptomatic CRC. Population awareness campaigns and urgent referrals are the main initiatives used to increase the early detection of symptomatic CRC [3, 4]. Despite efforts to reduce the time from the onset of first symptoms to diagnosis and after more than four decades of research on this subject, controversy remains about the relationship of the time from symptom onset to diagnosis with survival of patients with CRC [5–7].

Some indirect evidence suggested that a long diagnostic interval was associated with poor survival of these patients [7]. However, two systematic reviews that analyzed the relationship of the time from symptom onset to diagnosis with tumor stage and with patient survival found no significant associations [8, 9]. A more recent systematic review that included new studies published up to November 2013 [10] also did not resolve this issue. In particular, this more recent publication [10] identified some studies which reported that a long time from symptom onset to diagnosis was associated with poor survival; some studies which reported that a short diagnostic delay was associated with poor survival, and other studies that found no relationship between time from symptom onset to diagnosis and survival.

In 1994, Macguire et al. [11] proposed that the relationship between time from symptom onset to diagnosis and patient prognosis was nonlinear. In the last decade, some studies incorporated this new paradigm, and examined the nonlinear relationship of the time from symptom onset to diagnosis with cancer outcomes. These studies reported poor survival both in patients with short intervals from symptom onset to diagnosis, as well as in those with very long times from symptom onset to diagnosis [12–15]. However, some studies that used similar methodologies failed to find this association [16], often because they did not adequately control for potential confounders [17].

The aim of this study was to investigate the association of the time from symptom onset to diagnosis with survival in a cohort of 950 patients with CRC. This study assumed there was a nonlinear relationship between the time from symptom onset to diagnosis and survival, and adjusted for multiple confounders, such as tumor grade, symptoms, and presentation at an emergency department.

## Methods

This multicenter study with prospective follow-up examined patients in five regions of Spain (Aragón, Balearic Islands, Catalonia, Galicia, Valencia). The cohort of 950 consecutive patients with incident CRC was examined from September 2006 to September 2008 (DECCIRE Study) [18, 19]. Patients were identified from the pathology services of 9 public hospitals. Patients were excluded if they were younger than 18 years, had prevalent or recurrent CRC, had multiple tumors, were not registered with a general practitioner (GP), or were diagnosed in a private hospital. asymptomatic patients were also excluded (screening detected cases, incidental finding). The cohort of patients was followed up from 2010 to 2012 to assess survival status (DECCIRE II study). The detailed protocol of this study was previously published [20]. This study adheres to STROBE guidelines.

### Measurements

#### Data collection at cohort inception

Data were from patient interviews that were conducted by trained GPs and nurses immediately after diagnosis. These data included initial CRC symptoms, date of first presentation, perception of the seriousness of symptoms, help-seeking behavior, socio-demographic factors (age, sex, marital status, and level of education), and history of cancer in family members or acquaintances. Each patient was asked how long he/she felt unwell. If the patient remembered the exact date, that date was recorded; if the patient could not remember the exact date, then an approximate date was recorded. The first symptoms were the symptoms spontaneously reported by the patient without prompting by a GP or nurse. After recording the first symptoms and the date of onset, the interviewer asked each patient if he/she presented with any other symptoms on a checklist of 22 frequent CRC symptoms. For patients who were not interviewed, the date of first symptoms recorded in the primary health care record or the hospital record was used. Data from the hospital records included date of first symptoms (recorded at the first visit), tumor characteristics (grade, TNM stage, and location), and date of diagnosis (based on the date of the first histology report), and the presence of an intestinal occlusion. The first hospital service that evaluated the patient was classified as an emergency department or an outpatient service. The Charlson Comorbidity Index (CCI) at diagnosis was recorded based on comorbidities registered in clinical records. In addition, we collected the following treatment-related variables: resection (curative or palliative) and oncological treatment (chemotherapy and radiotherapy: before or after resection).

#### Data collection during follow up

During follow-up, survival time and mortality (including date and cause of death) were recorded using data from the Regional Mortality Registries.

### Statistical analysis

Survival time (all-cause mortality) was the date from diagnosis to the final outcome. The main predictor of survival was the time from symptom onset to diagnosis.

Time intervals are presented as medians and inter-quartile ranges (IQRs) and categorical variables as proportions. Patients were allocated into quartiles according to their times from symptom onset to diagnosis: 0–1.9 months (0–57 days), 1.91–4.20 months (57–127 days), 4.21–8.4 months (127–257 days), and more than 8.4 months (257–639 days). The relationship of the time from symptom onset to diagnosis with co-variables was assessed using the chi square test (categorical variables) and ANOVA (continuous variables). A relationship was considered significant if the associated P value was 0.05 or less. Bivariate and multivariate Cox regression analyses were used to analyze the relationship of the duration of diagnostic delay with mortality after adjusting for co-variables. Hazard ratios (HRs) for mortality and 95% confidence intervals (CIs) were calculated. Patients were censored if they were not in the mortality registry or did not have contact with a health service point during the prior 6 months. Predictors considered as prior potential confounders and those associated with the exposure (time from symptom onset to diagnosis) and the outcome (mortality) were included in the analysis. Multivariate regression models were fitted with adjustment for the colon and rectum jointly, and with separate adjustment for the colon and rectum. Final models were subjected to manual backward fitting using the likelihood-ratio test. Determination of Schoenfeld residuals and a log–log graph indicated there were no violations of the proportional hazards assumption. Tumor stage at diagnosis was considered an intermediate variable and was not included in the multivariate analysis.

Although the proportional hazard assumption was not violated, a nonlinear relationship between time from symptom onset to diagnosis and mortality was assumed. This approach allowed identification of a more flexible association between the time from symptom onset to diagnosis and mortality. Cubic spline regression analysis was performed using restricted cubic splines with four knots and 4.19 days (colon and rectum), 4.11 days (colon), and 4.26 days (rectum) as the reference points. A sensitivity analysis was also conducted measuring CRC -specific survival (please refer to Supplementary Tables S1, S2 and supplementary figures S1, S2 and S3. Also, we did an additional stage specific cubic spline regression analysis for overall mortality presented in Fig. 4 and for CRC-specific survival (Supplementary figure S4). Data were analyzed using STATA version 13 (Stata Corp, TX, USA).

## Results

There were 795 CRC patients who were symptomatic, 779 (97.9%) of them had data on the time from symptom onset to diagnosis, and 769 (96.7%) of them had complete follow-up data (In 10 cases we cannot confirm their situation as alive or dead). We initially analyzed the sociodemographic and clinical characteristics of patients diagnosed with CRC after presentation with symptoms (Table 1). The median age at diagnosis was 72 years (interquartile range [IQR]:62–78), most patients only completed elementary education, and there were more men (62.2%) than women (37.8%). The median time from symptom onset to diagnosis was 4.2 months (IQR: 1.9–8.4). Most tumors were in the colon (61.1%) and were grade I or II (89.3%). More than half of the patients had tumor stage II or III at diagnoses, and 19.4% had stage IV metastatic tumors. Most surgeries had a curative intention (92%), 30% did not receive oncological treatment, 48% received chemotherapy,and 21% both radiotherapy and chemotherapy. The median follow-up time was 57.9 months (IQR: 23.6–67.2). The total overall survival rate was 68.3% at 3 years and 57.3% at 5 years, and the mean survival time was 45.0 months (95%CI: 43.6–46.3). There was no information about CRC-specific deaths in 196 cases. CRC-specific mortality was 67.2% at 3 years and 65.5% at 5 years.

**Table 1:** Association of sociodemographic factors, clinical factors, and time from symptom onset to diagnosis, with 5-year overall survival in patients with colorectal cancer

	Total (*n* = 779) n/N (%)	Deaths at 5 years follow-up (*n* = 347)	HR (95% CI)	*P* value	Mortality rate n/N (%)
Time from first symptom presentation to diagnosis (in months)	4.2 (1.9–8.4)	3.7 (1.6–7.5)	0.97 (0.96–0.99)	0.001	NA
First symptom presentation to diagnosis (quartiles)					
1^st^ quartile (< = 1.9 months)	195/779 (25.0)	96/347 (27.7)	1		96/193 (49.7)
2^nd^ quartile (1.9–4.2 months)	197/779 (25.3)	103/347 (29.7)	1.00 (0.76–1.33)	0.948	103/196 (52.6)
3^rd^ quartile (4.2–8.4 months)	192/779 (24.2)	71/347 (20.5)	0.67 (0.49–0.91)	0.008	71/187 (38.0)
4^rd^ quartile (> 8.4 months)	195/779 (24.6)	77/347 (22.2)	0.72 (0.53–0.97)	0.034	77/193 (39.9)
Age median (IQR) years	72 (62 -78)	75 (66–80)	1.04 (1.03–1.05)	< 0.001	NA
Sex					
Men	482/775 (62.2)	227/346 (65.6)	1		227/474 (47.9)
Women	293/775 (37.8)	119/346 (34.4)	0.87 (0.71–1.07)	0.184	119/291 (40.9)
Level of education					
Primary	504/720 (70.0)	212/296 (71.6)	1		212/497 (42.7)
High school	170/720 (23.6)	64/296 (21.6)	0.87 (0.66–1.15)	0.326	64/168 (38.1)
University	46/720 (6.4)	20/296 (6.8)	1.03 (0.65–1.63)	0.901	20/455 (44.4)
Tumor Stage					
0-I	137/701 (19.5)	32/305 (10.5)	1		32/134 (23.9)
II	226/701 (32.2)	71/305 (23.3)	1.39 (0.93–2.10)	0.101	71/225 (31.6)
III	202/701 (28.8)	84/305 (27.5)	1.98 (1.33–2.94)	0.001	84/200 (42.0)
IV	136/701 (19.4)	118/305 (38.7)	8.15 (5.54–12.0)	< 0.001	118/135 (87.4)
Localization					
Colon	470/769 (61.1)	204/340 (60.0)	1		204/464 (44.0)
Rectum	299/769 (38.9)	136/340 (40.0)	1.04 (0.84–1.30)	0.678	136/295 (46.1)
Tumor grade					
Grade I	166/726 (22.9)	67/326 (20.5)	1		66/163 (40.5)
Grade II	482/726 (66.4)	214/326 (65.6)	1.22 (0.93–1.61)	0.138	210/475 (44.2)
Grade III/IV	59/726 (8.1)	34/326 (10.4)	2.04 (1.35–3.09)	0.001	33/59 (55.9)
Ungraded	19/726 (2.6)	11/326 (3.4)	-	-	11/19 (57.9)
Intestinal obstruction					
No	659/764 (86.3)	277/342 (81.0)	1		277/651 (42.5)
Yes	105/764 (13.7)	65/342 (19.0)	1.90 (1.45–2.48)	< 0.001	65/104 (62.5)
Emergency presentation					
No	416/772 (53.9)	157/349 (45.8)	1		157/407 (38.6)
Yes	356/772 (46.1)	189/349 (54.2)	1.58 (1.28–1.95)	< 0.001	186/355 (52.4)
Perception of seriousness					
Not serious	458/668 (68.6)	197/280(70.4)	1		195/455 (42.9)
Quite serious	162/668 (24.3)	66/280 (23.6)	1.05 (0.80–1.38)	0.745	66/157 (42.0)
Very serious	48/668 (7.2)	17/280 (6.1)	0.78 (0.47–1.28)	0.326	17/48 (35.4)
Help seeking behavior					
Wait	184/671 (27.4)	75/277 (27.1)	1		201/480 (41.9)
Visit doctor	487/671 (72.6)	202/277 (72.9)	1.05 (0.81–1.37)	0.705	75/182 (41.2)
Charlson index					
Median (IQR)	1 (0–2)	1 (0–2)	1.18 (1.11–1.27)	< 0.001	NA
First Symptoms presentation					
Abdominal Pain					
No	517/711 (72.7)	220/292 (75.3)	1		220/509 (43.2)
Yes	194/711 (27.3)	72/292 (24.7)	0.84 (0.65–1.10)	0.207	72/192 (37.5)
Tenesmus					
No	651/711 (91.6)	267/292 (91.4)	1		25/58 (43.1)
Yes	60/711 (8.4)	25/292 (8.6)	1.04 (0.69–1.57)	0.848	267/643 (41.5)
Rectal bleeding					
No	427/711 (60.1)	191/292 (65.4)	1		191/421 (45.4)
Yes	284/711 (39.9)	101/292 (34.6)	0.71 (0.56–0.91)	0.008	101/280 (36.1)
Constipation					
No	598/711 (84.1)	232/292 (79.5)	1		60/111 (54.1)
Yes	113/711 (15.9)	60/292 (20.5)	1.44 (1.08–1.91)	0.015	232/590 (39.3)
Weight loss					
No	654/711 (92.0)	264/292 (90.4)	1		264/645 (40.9)
Yes	57/711 (8.0)	28/292 (9.6)	1.31 (0.89–1.93)	0.170	28/56 (50.0%)
Anorexia					
No	662/711 (96.1)	267/292 (91.4)	1.53 (1.01–2.31)		267/654 (40.8)
Yes	49/711 (6.9)	25/292 (8.6)	1	0.042	25/47 (53.2)
Abdominal mass					
No	696/711 (97.9)	285/292 (97.6)	1		285/686 (41.5)
Yes	15/711 (2.1)	7/292 (2.4)	1.15 (0.54–2.43)	0.701	7/15 (46.7)
Tiredness					
No	614/711 (86.4)	267/292 (84.2)	1		246/607 (40.5)
Yes	97/711 (13.6)	46/292 (15.8)	1.31 (0.95–1.79)	0.092	46/94 (48.9)
Treatment					
Surgery					
Curative Resection	611/664 (92.0)	231/277 (83.4)	1	< 0.001	231/605 (38.2)
Palliative Resection	53/664 (7.98)	46/277 (16.6)	4.88 (3.53–6.75)		46/53 (86.8)
Oncological treatment					
No	160/527 (30.4)	51/220 (23.2)	1		
Chemotherapy	254/527 (48.2)	128/220 (58.2)	1.71 (1.23–2.36)		
Chemotherapy/Radiotherapy	113/527 (21.4)	41/220 (18.6)	1.10 (0.73–1.66)		

We also performed a bivariate Cox regression analysis to determine the relationship of different factors, including time from symptom onset to diagnosis, with 5-year overall survival. In the unadjusted analysis, times from symptom onset to diagnosis of 4.2 to 8.4 months and more than 8.4 months were associated with a decreased risk of mortality. Patients who were older, had an advanced stage tumor, had a grade III or IV tumor, presented at an emergency department, had an intestinal obstruction, had a higher CCI, had anorexia and constipation as first symptoms and those treated with chemotherapy or radiotherapy-chemotherapy had an increased risk of death. Rectal bleeding as a first symptom was associated with a decreased risk of mortality. There were no significant associations of death with sex, level of education, tumor localization, perception of symptom severity, help-seeking behavior, and other symptoms and type of resection.

We then examined the relationship of different patient characteristics with the duration of the diagnostic delay (Table 2). The results indicated the diagnostic delay was longer for patients who were older, female, perceived their symptoms as not serious, or had weight loss as a first symptom. The time from symptom onset to diagnosis was shorter for those who had an intestinal obstruction, presented at an emergency department, exhibited help-seeking behavior, had abdominal pain, and had constipation. There were no significant associations between time from symptom onset to diagnosis and other factors, including level of education, history of cancer in the family and acquaintances, other comorbidities, other first symptoms, tumor grade, tumor stage, and tumor location.

**Table 2:** Association of sociodemographic factors, clinical factors, and initial symptoms with time from symptom onset to diagnosis in patients with colorectal cancer

	1st quartile < = 1.9 month	2nd quartile 1.9–4.2 month	3rd quartile 4.2–8.4 month	4rd quartile > 8.4 months	*P* value
Age median (IQR) years	71 (63.5–78)	72 (63–77)	69 (59.7–77)	74 (62–79)	0.038
Sex: Women	65/193 (33.7)	61/197 (31.0)	88/192 (45.8)	79/193 (40.9)	0.010
Level of education:					
Primary	120/175 (69.0)	128/183 (69.9)	123/181 (68.0)	133/182 (73.1)	
High school	39/175 (22.4)	45/183 (24.6)	46/181 (25.4)	40/182 (22.0)	
University	15/175 (8.6)	10/183 (5.5)	12/181 (6.6)	9/182 (4.9)	0.780
Tumor Stage					
0-I	30/176 (17.0)	31/181 (17.1)	35/172 (20.3)	41/172 (23.8)	
II	65/176 (36.9)	54/181 (29.8)	61/172 (35.5)	46/182 (26.7)	
III	46/176 (26.1)	56/181 (30.9)	49/172 (28.5)	51/182 (29.7)	
IV	35/176 (19.9)	40/181 (22.1)	27/172 (15.7)	34/182 (19.8)	0.421
Localization					
Colon	115/194 (59.3)	125/197 (63.5)	113/192 (58.9)	120/195 (61.5)	
Rectum	79/194 (40.7)	72/197 (36.5)	79/192 (41.1)	75/195 (38.5)	0.782
Tumor grade					
Grade I	37/174 (21.3)	46/192 (24.0)	39/179 (21.8)	44/181 (24.3)	
Grade II	119/174 (68.4)	123/192 (64.1)	117/179 (65.4)	123/181 (68.0)	
Grade III/IV	12/174 (6.9)	19/192 (9.9)	18/179 (10.1)	10/181 (5.6)	
Ungraded	6/174 (3.4)	4/192 (2.1)	5/179 (2.8)	4/181 (2.2	0.825
Intestinal obstruction	42/192 (21.9)	22/195 (11.3)	24/187 (12.8)	17/190 (8.9)	0.001
Emergency presentation	130/193 (67.4)	89/197 (45.2)	73/189 (38.6)	64/193 (33.2)	< 0.001
Perception of seriousness					
Not serious	102/157 (65.0)	105/173(60.7)	127/167 (76.0)	124/171 (72.5)	
Quite serious	37/157 (23.6)	50/173(28.9)	31/167 (18.6)	44/171 (25.7)	0.001
Very serious	18/157 (11.5)	18/173 (10.4)	9/167 (5.4)	3/171 (1.8)	
Help seeking behavior					
Visit doctor	129/156 (82.7)	125/170 (73.5)	125/176 (71.0)	108/169 (63.9)	
Wait	27/156 (17.3)	45/170 (26.5)	51/176 (29.0)	61/169 (36.1)	0.002
Charlson index					
Median (IQR)	1 (0–2)	1 (0–2)	0 (0–2)	1 (0–1)	0.237
First symptoms presentations					
Abdominal Pain	59/174 (33.9)	46/179 (25.7)	52/177 (29.4)	37/181 (20.4)	0.033
Tenesmus	19/174 (10.9)	12/179 (6.7)	17/177 (9.6)	12/181 (6.6)	0.360
Rectal bleeding	75/174 (43.1)	72/179 (40.2)	66/177 (37.3)	71/181 (39.2)	0.732
Constipation	35/174 (20.1)	29/179 (16.2)	31/177 (17.5)	18/181 (9.9)	0.058
Weight loss	14/174 (8.0)	15/179 (8.4)	21/177 (11.9)	7/181 (3.9)	0.050
Anorexia	15/174 (8.6)	9/179 (5.0)	18/177 (10.2)	7/181 (3.9)	0.062
Abdominal mass	2/174 (1.1)	3/179 (25.2)	5/177 (2.8)	5/181 (2.8)	0.687
Tiredness	18/174 (10.3)	25/179 (14.0)	28/177 (15.8)	26/181 (14.4)	0.491

We performed multivariate Cox regression analysis to identify factors significantly associated with cancer of the colon or rectum (Model-1), cancer of the colon alone (Model-2), and cancer of the rectum alone (Model-3; Table 3). The results indicated that age (all models), tumor location (Model-1), tumor grade (all models), intestinal obstruction (Model-1 and -2), emergency presentation (Model-1 and -2), and Charlson index (all models) were associated with an increased risk of 5-year mortality. Rectal bleeding (Model-1) was inversely associated with 5-year mortality. Notably, the time from symptom onset to diagnosis was not significantly associated with 5-year mortality.

**Table 3:** Multivariate adjusted Cox proportional hazard models of the relationship of 5-year overall mortality with time from symptom onset to diagnosis and other factors in patients with cancer of the colon or rectum, colon alone, and rectum alone

	Colon and Rectum Model I	Colon Model II	Rectum Model III	
	HR 95% CI	HR 95% CI	HR 95% CI	
Total diagnostic lag time (quartiles)				
1st quartile (< = 1.9 months)	1	1	1	
2nd quartile (1.9–4.2 months)	1.24 (0.80–1.94)	1.10 (0.60–2.04)	1.80 (0.89–3.65)	
3rd quartile (4.2–8.4 months)	0.80 (0.48–1.34)	1.06 (0.54–2.07)	0.63 (0.27–1.49)	
4rd quartile (> 8.4 months)	1.01 (0.62–1.66)	1.09 (0.55–2.16)	1.42 (0.65–3.11)	
Age median (IQR) years	1.03 (1.02–1.06)	1.04 (1.01–1.06)		1.05 (1.02–1.09)
Sex				
Men	1	1		1
Women	0.73 (0.50–1.05)	0.67 (0.42–1.08)		0.67 (0.35–1.29)
Localization				
Colon	1			
Rectum	1.44 (0.92–2.26)	-		-
Tumor grade				
Grade I	1	1		1
Grade II	1.52 (0.96–2.42)	1.30 (0.72–2.33)		2.86 (1.23–6.67)
Grade III/IV	2.38 (1.20–4.62)	1.44 (0.60–3.43)		7.61 (2.47–23.33)
Ungraded	1.19 (0.43–3.29)	0.63 (0.08–4.96)		3.34 (0.82–13.70)
Intestinal obstruction				
No	1	1		1
Yes	1.78 (1.11–2.84)	1.32 (0.77–2.27)		14.61 (4.76–44.84)
Emergency presentation				
No	1	1		1
Yes	1.44 (0.99–2.08)	1.54 (0.93–2.56)		1.66 (0.91–3.03)
Charlson index				
Median (IQR)	1.15 (1.01–1.33)	1.12 (0.93–1.36)		1.34 (1.05–1.71)
Rectal bleeding				
No	1	1		1
Yes	0.74 (0.50–1.11)	0.52 (0.28–0.97)		1.26 (0.70–2.24)
Treatment				
Surgery				
Curative Resection	1	1		1
Palliative Resection	4.54 (2.83–7.29)	3.91 (2.06–7.41)		6.58 (2.84–15.23)
Oncological treatment				
No	1	1		1
Chemotherapy	2.01 (1.26–3.20)	1.87 (1.07–3.28)		2.34 (0.90–6.07)
Chemotherapy/Radiotherapy	1.68 (0.92–3.07)	-		2.99 (1.27–7.04)

**Fig. 1:**
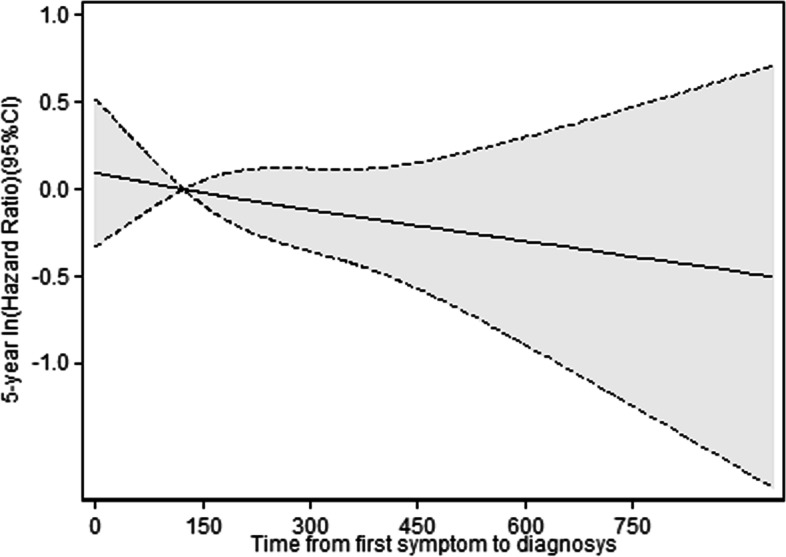
Cubic spline regression analysis of the relationship of time from symptom onset to diagnosis with 5-year overall survival in patients with cancer of the colon or rectum

**Fig. 2:**
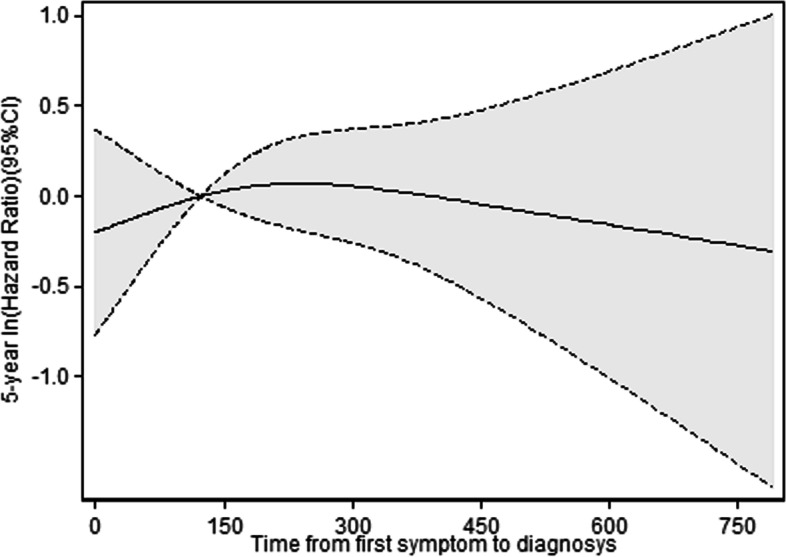
Cubic spline regression analysis of the relationship of time from symptom onset to diagnosis with 5-year overall survival in patients with cancer of the colon

**Fig. 3:**
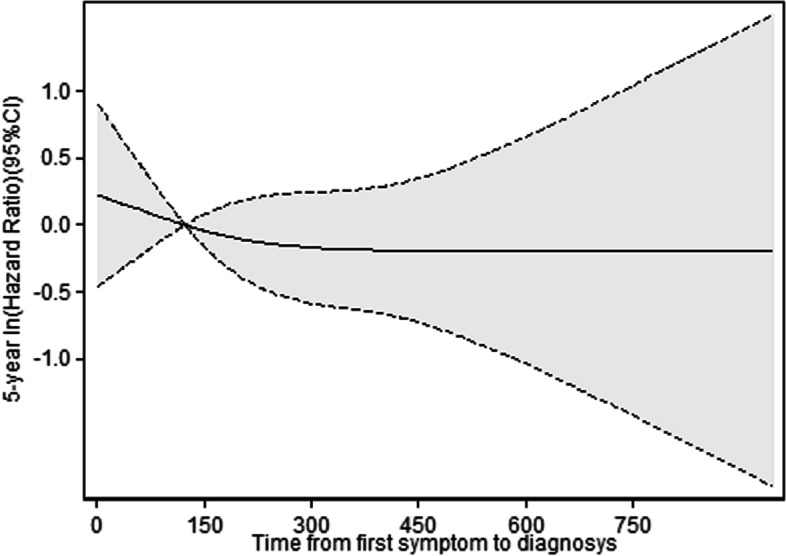
Cubic spline regression analysis of the relationship of time from symptom onset to diagnosis with 5-year overall survival in patients with cancer of the rectum

We also performed cubic spline regression analysis of the relationship of the duration of diagnostic delay with 5-year overall survival in patients with cancer of the colon or rectum (Fig. 1), cancer of the colon alone (Fig. 2) and cancer of the rectum alone (Fig. 3).

**Fig. 4:**
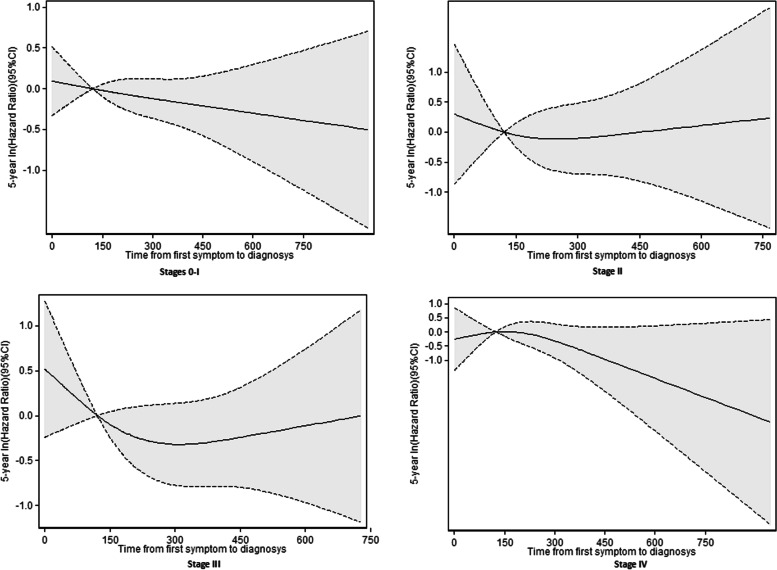
Stage specific cubic spline regression analysis of the relationship of time from symptom onset to diagnosis with 5-year overall survival in patients with colorectal cancer

These models adjusted for age, sex, tumor location, tumor grade, emergency presentation, intestinal obstruction, comorbidities, and symptoms. In each case, there was an inverse association between time from symptom onset to diagnosis and 5-year survival, but all the confidence intervals were very wide and none of these associations were statistically significant. We also found an overall inverse association in the analysis of stage specific spline curves for the adjusted model (Fig. 4).

We performed a sensitivity analysis of the association of time to diagnosis and 5-year CRC-specific mortality and obtained similar results (Supplementary material Tables S1 and S2; Figures S1, S2, S3 and S4).

## Discussion

The results of our multivariate adjusted Cox proportional hazard models indicated that a longer time from symptom onset to diagnosis was not associated with increased hazard of mortality in patients with symptomatic CRC. Our cubic spline analysis, which considered nonlinearities, indicated an inverse association between the duration of diagnostic delay and survival; however, this association was not statistically significant. Other CRC studies that examined the time from symptom onset to diagnosis and also used restricted cubic spline analyses reported similar results [16, 17].

In contrast, Tørring et al. [14] found a U-shaped relationship between the diagnostic interval and mortality in five common cancers. In particular, for patients attended by a GP due to alarming symptoms, the risk of dying decreased as the diagnostic interval increased up to 5 weeks, and the risk then increased. These results were contrary to those for patients with vague or non-specific symptoms, although that relationship was not significant [12]. Importantly, Tørring et al. [14] found similar U-shaped relationships for patients with melanoma, breast cancer, prostate cancer, and lung cancer, but only for those with alarming symptoms [14]. However, neither of these studies adjusted for potential confounders.

The ‘waiting time paradox’, first described in a clinical setting by Crawford et al. [21], refers to the inverse association of survival with time until onset of treatment or diagnosis. A likely explanation of this paradox is that the nature of the disease affects the delay, and delay is therefore a confounder. In other words, when patients first present with symptoms, clinicians can reliably identify patients with more severe disease and then administer further diagnostic testing or treatment to these patients. Although we were able to adjust for some potential confounders, such as tumor grade, emergency presentation, symptoms, and comorbidities, it is possible that residual and unmeasured confounders contributed to the waiting time paradox. Furthermore, the relationship between cancer symptoms and stage is not straightforward and rarely linear; symptoms are often totally absent in patients with late-stage cancers. There is also high heterogeneity in the stage of CRC when the initial symptoms appear. We previously proposed that the CRC stage when symptoms first appear is an important and unknown confounder, reasonably the time to diagnosis and survival time would be shorter if symptoms of CRC appeared at stage III or IV [22] and this made it difficult for us to ascertain whether the time duration between symptom onset and diagnosis influenced survival. Majumbar et al. suggested that the symptomatic phase in some patients with CRC may be a very late event during the course of this disease, and that a difference of several months in the time from symptom onset to diagnosis may not have a significant effect on outcome [23]. This interpretation is consistent with our findings.

In the last decade, researchers examining the relationship of the time from symptom onset to diagnosis with survival have used more rigorous study designs in an effort to better define diagnosis-treatment intervals, as recommended by Arhi et al. [24]. Thus, recent studies of this topic have used larger sample sizes, imposed fewer restrictions on patient inclusion, and considered potential confounders. Nevertheless, comparisons of studies are still difficult because there are significant differences in the methods, patient characteristics, and inclusion/exclusion criteria, i.e., focusing on patients admitted to emergency departments or older patients [21, 24, 25]. Many studies have measured the time from symptom onset to diagnosis [16, 21, 26], as in the present study, but others measured the time from initial presentation to diagnosis [12–14, 17, 23, 25, 27]. Studies that measured the total diagnostic interval reported no association between long diagnostic interval and risk of death [16, 26, 28] but Pita et al. found that a shorter lag time was associated with lower survival in patients with rectal cancer [16]. However, using the interval from onset of symptoms to diagnosis, let have a broad idea of the diagnostic interval as it includes patient delay that is, from symptoms onset to presentation to a health professional. This time is relevant as it could account for 20% to 40% of the total diagnostic interval [19, 27]. Interventions centered on health professionals and organizations are important, but it is also necessary to increase interventions that increase population awareness of cancer symptoms and address help-seeking behaviors.

### Limitations and strengths

The present study had some limitations. First, as in all studies based on interview data, there was a risk of recall bias. Because we interviewed most patients after surgery (an average of 47 days after diagnosis) they may have had difficulties recalling the exact time of symptom onset, particularly if the symptoms were mild or non-specific, and this could have led to information misclassification. A patient with poor prognosis or with symptoms that appeared more recently might also provide more reliable information regarding the date of presentation and symptoms. However, any source of information is susceptible to misclassification. A previous study compared how the date of symptom onset from three sources affected the total diagnostic interval, and demonstrated that data were affected by information bias and non-random classification [22]. We measured all-cause mortality from the date of diagnosis to death or censoring. Some authors suggested that this might cause lead-time bias, but sensitivity analysis that measured the time from diagnosis at presentation led to similar results [17]. We found similar results when analyzing all-cause mortality and CRC-specific mortality. Pruitt et al. [29] found divergences between all-cause and CRC-specific death when measuring the relationship between time to diagnosis or treatment and survival. Despite high variation of time to treatment between CRC patients [18, 29], we did not measure the relationship of treatment delay and survival both as an independent interval or added to the diagnosis interval. Again, there are controversial results in studies that measured treatment delay and mortality. Some authors found that shorter treatment delays had the higher odds of all-cause [29] but not for CRC-specific death. Others observed that the risk of death significantly increased in those patients with treatment delay >  = 30 days [30]. Nevertheless, analogous results have been found with those of Murchie et al. [17] where after adjusting for multiple confounders no relationship between time of presentation to treatment was observed.

One of the strengths of our study is that we included incident cases and calculated the interval from the onset of symptoms until diagnosis, similar to the studies of Pita et al. [16] and Singh et al. [25]. As these authors mentioned, studies that measure the diagnosis time independently of the location of diagnosis (out-patient clinic, emergency department, etc.) may have more external validity because they include all types of patients. In comparison, studies that only examine patients who visited primary care centers probably have more homogenous patient populations and greater internal validity, but they may have excluded 20 to 30% of the patients who were diagnosed with CRC at emergency departments or other outpatient referral services [19, 31, 32]; the different characteristics of these groups could be related to differences outcome. Several studies showed that CRC patients who bypassed their GPs were diagnosed earlier [33] and had poorer outcomes [34]. The 2011 study of Tørring et al. [12] found that 56% of the patients in which the GP was not involved in the diagnosis were admitted to an emergency room. Moreover, the present study examined CRC incident cases in different public tertiary hospitals and general hospitals, and thus considered a wide variety of patients whose diagnoses were by a wide variety of clinicians. We had complete data for most patients; these data were about 90% complete for tumor stage and grade and more than 96% complete for follow up and diagnostic delay. Furthermore, the diverse variables that we examined enabled adjustment for potential confounding, as in other studies of this topic. We included variables that were significantly associated with risk of death in the bivariate analysis. Variables such as grade, emergency department presentation, and intestinal occlusion may be markers of fast-growing tumors and could potentially contribute to the account for a ‘waiting time paradox’ (poorer prognosis in patients with shorter times from symptom onset to diagnosis). Moreover, adjustment for age and comorbidities is essential because they are related to patient mortality and time from symptom onset to diagnosis [35]. We found treatment as factors associated with survival. However, the treatment the patient received is closely related to the stage at diagnosis. We did not adjust by tumor stage because the relationship between diagnostic delay, tumor stage, and survival is complex. This relationship is often considered an intermediate factor between time from symptom onset to diagnosis and survival because it is on the causal pathway that connects time from symptom onset to diagnosis and survival. The stage specific analysis did not show significant association between length of diagnosis and survival.

## Conclusions

Our results provided no evidence that the time from symptom onset to diagnosis affected the prognosis of patients with symptomatic CRC. These results are similar to those of several previous studies, although some other studies reported contrary results. We suggest that the most important determinant affecting the diagnostic duration is the biological profile of the tumor and its consequent clinical effects.

## Supplementary Information

**Additional file 1.** Supplementary tables

**Additional file 2.** Supplementary figures 1-3

**Additional file 3.** Supplementary fig 4

## Data Availability

The datasets used and analysed during the current study are available from the corresponding author on reasonable request.

## Abbreviations

CRC: Colorectal cancer
GP: General practitioner
CCI: The Charlson Comorbidity Index
IQRs: Inter-quartile ranges
CIs: Confidence intervals
HRs: Hazard ratios
