# Supplementary material for: Relationship between time from symptom’s onset to diagnosis and prognosis in patients with symptomatic colorectal cancer
Source: BMC Cancer. 2022 Aug 22;22:910. doi: 10.1186/s12885-022-09990-7 (PMC9394014; doi:10.1186/s12885-022-09990-7)
Supplement: Supplementary file 1 — Additional file 1. Supplementary tables [file 12885_2022_9990_MOESM1_ESM.docx]

**Supplementary Table 1.- Association of sociodemographic factors, clinical factors, and time from symptom onset to diagnosis, with 5-year CRC-specific survival in patients with colorectal cancer.**

|  | Total (n=779)  n/N (%) | Deaths at 5 years follow-up  (n=262) | HR (95% CI) | P value | Mortality rate  n/N (%) |
| --- | --- | --- | --- | --- | --- |
| Time from first symptom presentation to diagnosis (in months) | 4.2 (1.9-8.4) | 3.6 (1.6-7.5) | 0.97 (0.95-0.99) | 0.007 | NA |
| Firs symptom presentation to diagnosis (quartiles)  1^st^ quartile (<=1.9 months)  2^nd^ quartile (1.9-4.2 months)  3^rd^ quartile (4.2-8.4 months)  4^rd^ quartile (>8.4 months) | 195/779 (25.0)  197/779 (25.3)  192/779 (24.2)  195/779 (24.6) | 76/263 (28.9)  75/263 (28.5)  51/263 (19.4)  61/263 (23.2) | 1  0.92 (0.67-1.27)  0.60 (0.42-0.86)  0.72 (0.52-1.01) | 0.634  0.005  0.058 | 76/191 (39.8)  75/194 (38.7)  51/185 (27.6)  61/192 (31.8) |
| Age median (IQR) years | 72 (62 -78) | 73 (63-79) | 1.02 (1.01-1.03) | <0.001 | NA |
| Sex:  Men  Women | 482/775 (62.2)  293/775 (37.8) | 168/262 (64.1)  94/262 (35.9) | 1  0.89 (0.69-1.14) | 0.364 | 168/470 (35.7)  94/288 (32.6) |
| Level of education:  Primary  High school  University | 504/720 (70.0)  170/720 (23.6)  46/720 (6.4) | 164/227 (72.5)  48/227 (21.1)  15/227 (6.6) | 1  0.83 (0.60-1.15)  0.99 (0.58-1.69) | 0.261  0.987 | 212/497 (42.7)  64/168 (38.1)  20/455 (44.4) |
| Tumor Stage  0-I  II  III  IV | 137/701 (19.5)  226/701 (32.2)  202/701 (28.8)  136/701 (19.4) | 17/234 (7.3)  38/234 (16.2)  66/234 (28.2)  113/234 (48.3) | 1  1.37 (0.77-2.42)  2.97 (1.74-5.06)  14.7 (8.8-24.6) | 0.283  <0.001  <0.001 | 17/134 (12.7)  38/224 (17.0)  66/197 (33.5)  113/134 (84.3) |
| Location  Colon  Recto | 470/769 (61.1)  299/769 (38.9) | 148/257 (57.6)  109/257 (42.4) | 1  1.16 (0.91-1.40) | 0.220 | 148/460 (32.2)  109/292 (37.3) |
| Tumor grade  Grade I  Grade II  Grade III/IV  Ungraded | 166/726 (22.9)  482/726 (66.4)  59/726 (8.1)  19/726 (2.6) | 44/240 (18.3)  162/240 (67.5)  24/240 (10.0)  10/240 (4.2) | 1  1.35 (0.97-1.89)  2.12 (1.29-3.49)  - | 0.076  0.003  - | 44/162 (27.2)  162/472 (34.3)  24/57 (42.1)  10/19 (52.6) |
| Intestinal obstruction  No  Yes | 659/764 (86.3)  105/764 (13.7) | 210/258 (81.4)  48/258 (18.6) | 1  1.86 (1.36-2.55) | <0.001 | 210/644 (32.6)  48/104 (46.1) |
| Emergency presentation  No  Yes | 416/772 (53.9)  356/772 (46.1) | 119/259 (45.9)  140/259 (54.0) | 1  1.53 (1.20-1.95) | 0.001 | 119/404 (29.5)  140/351 (39.9) |
| Perception of seriousness  Not serious  Quite serious  Very serious | 458/668 (68.6)  162/668 (24.3)  48/668 (7.2) | 151/212(71.2)  48/212 (22.6)  13/212 (6.1) | 1  0.99 (0.72-1.37)  0.78 (0.44-1.38) | 0.982  0.396 | 151/450 (33.6)  48/156 (30.8)  13/48 (27.1) |
| Help seeking behavior  Wait  Visit doctor | 184/671 (27.4)  487/671 (72.6) | 64/211 (30.3)  147/211 (69.7) | 1  0.92 (0.68-1.24) | 0.588 | 64/180 (35.6)  147/476 (30.9) |
| Charlson index  Median (IQR) | 1 (0-2) | 1 (0-2) | 1.15 (1.06-1.27) | 0.002 | NA |
| First Symptoms presentation |  |  |  |  |  |
| Abdominal Pain  No  Yes | 517/711 (72.7)  194/711 (27.3) | 166/223 (74.4)  57/223 (25.6) | 1  0.87 (0.65-1.18) | 0.380 | 166/504 (32.9)  57/191 (29.8) |
| Tenesmus  No  Yes | 651/711 (91.6)  60/711 (8.4) | 202/223 (90.6)  21/223 (9.4) | 1  1.20 (0.76-1.88) | 0.428 | 202/637 (31.7)  21/58 (36.2) |
| Rectal bleeding  No  Yes | 427/711 (60.1)  284/711 (39.9) | 149/223 (66.8)  74/223 (33.2) | 1  0.67 (0.51-0.89) | 0.005 | 149/419 (35.6)  74/276 (26.8) |
| Constipation  No  Yes | 598/711 (84.1)  113/711 (15.9) | 173/223 (77.6)  50/223 (22.4) | 1  1.61 (1.17-2.20) | 0.003 | 60/111 (54.1)  232/590 (39.3) |
| Weight loss  No  Yes | 654/711 (92.0)  57/711 (8.0) | 201/223 (90.1)  22/223 (9.9) | 1  1.32 (0.86-2.06) | 0.205 | 201/639 (31.5)  22/56 (39.3) |
| Anorexia  No  Yes | 662/711 (96.1)  49/711 (6.9) | 201/223 (90.1)  22/223 (9.9) | 1  1.79 (1.15-2.79) | 0.009 | 201/648 (31.0)  22/47 (46.8) |
| Abdominal mass  No  Yes | 698/711 (98.2)  13/711 (1.8) | 217/223 (97.3)  6/223 (2.7) | 1  1.55 (0.69-3.49) | 0.290 | 217/682 (31.8)  6/13 (46.1) |
| Tiredness  No  Yes | 614/711 (86.4)  97/711 (13.6) | 188/223 (84.3)  35/223 (15.7) | 1  1.30 (0.91-1.87) | 0.147 | 188/601 (31.3)  35/94 (37.2) |
| Treatment |  |  |  |  |  |
| Surgery  Curative Resection  Palliative Resection | 611/664 (92.0)  53/664 (7.98) | 163/204 (79.9)  41/204 (20.1) | 1  6.32 (4.47-8.95) | <0.001 | 163/600 (27.2)  41/53 (77.3) |
| Oncologic treatment  No  Chemotherapy  Chemotherapy/Radiotherapy | 160/527 (30.4)  254/527 (48.2)  113/527 (21.4) | 34/186 (18.3)  116/186 (62.4)  36/186 (19.3) | 1  2.31 (1.58-3.39)  1.45 (0.91-2.32) | <0.001  0.121 | 34/157 (21.7)  116/250 (49.4)  36/111 (32.4) |

**Supplementary Table 2.- Multivariate adjusted Cox proportional hazard models of the relationship of 5-year CRC-specific mortality with time from symptom onset to diagnosis and other factors in patients with cancer of the colon or rectum, colon alone, and rectum alone.**

|  |  | | | Colon and Rectum  Model I | | | Colon  Model II | Rectum  Model III |
| --- | --- | --- | --- | --- | --- | --- | --- | --- |
|  |  | | | | HR 95% CI | | HR 95% CI | HR 95% CI |
| Total diagnostic lag time (quartiles)  1st quartile (<=1.9 months)  2nd quartile (1.9-4.2 months)  3rd quartile (4.2-8.4 months)  4rd quartile (>8.4 months) | | |  | | | 1  1.29 (0.79-2.12)  0.84 (0.47-1.50)  1.04 (0.60-1.81) | 1  1.13 (0.58-2.22)  1.00 (0.47-2.14)  1.18 (0.56-2.46) | 1  1.91 (0.91-4.00)  0.68 (0.27-1.74)  1.41 (0.59-3.39) |
| Age median (IQR) years | | |  | | | 1.03 (1.02-1.06) | 1.03 (1.01-1.06) | 1.05 (1.01-1.08) |
| Sex  Men  Women | | |  | | | 1  0.83 (0.56-1.23) | 1  0.76 (0.45-1.26) | 1  0.89 (0.45-1.75) |
| Localization  Colon  Rectum |  | | | | | 1  1.60 (0.96-2.64) | - | - |
| Tumor grade  Grade I  Grade II  Grade III/IV  Ungraded |  | | | | | 1  1.55 (0.92-2.61)  2.36 (1.13-4.91)  2.47 (0.52-11.70) | 1  1.30 (0.69-2.51)  1.15 (0.42-3.10)  0.60 (0.05-4.93) | 1  3.21 (1.21-8.47)  12.0 (3.61-40.09)  3.97 (0.60-26.11) |
| Intestinal obstruction  No  Yes |  | | | | | 1  2.25 (1.36-3.72) | 1  1.65 (0.91-3.00) | 1  10.26 (3.52-29.90) |
| Emergency presentation  No  Yes |  | | | | | 1  1.21 (0.80-1.84) | 1  1.35 (0.76-2.38) | 1  1.52 (0.82-2.93) |
| Charlson index  Median (IQR) |  | | | | | 1.04 (0.87-1.24) | 1.01 (0.81-1.28) | 1.15 (0.86-1.53) |
| Rectal bleeding  No  Yes |  | | | | | 1  0.68 (0.43-1.06) | 1  0.49 (0.24-0.99) | 1  1.19 (0.63-2.23) |
| Surgery  Curative Resection  Palliative Resection | | | | | | 1  5.19 (3.14-8.60) | 1  4.52 (2.35-8.71) | 1  6.97 (2.79-17.37) |
| Oncologic treatment  No  Chemotherapy  Chemotherapy/Radiotherapy | |  | | | | 1  2.91 (1.65-5.15)  2.36 (1.68-4.79) | 1  2.92 (0.46-5.84)  - | 1  3.22 (1.08-9.59)  3.31 (1.21-9.00) |
