# Supplementary material for: Relationship between time from symptom’s onset to diagnosis and prognosis in patients with symptomatic colorectal cancer
Source: BMC Cancer. 2022 Aug 22;22:910. doi: 10.1186/s12885-022-09990-7 (PMC9394014; doi:10.1186/s12885-022-09990-7)
Supplement: Supplementary file 2 — Additional file 2. Supplementary figures 1-3 [file 12885_2022_9990_MOESM2_ESM.docx]

**
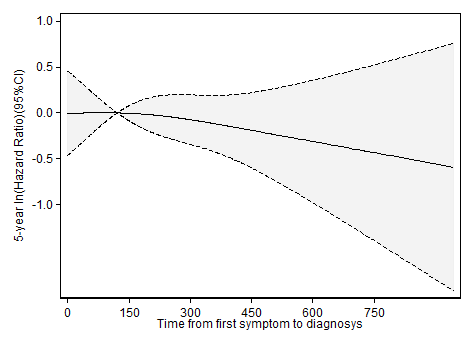
**

**Supplementary figure 1.-Cubic spline regression analysis of the relationship of time from symptom onset to diagnosis with 5-year CRC-specific survival in patients with colorectal cancer.**

**
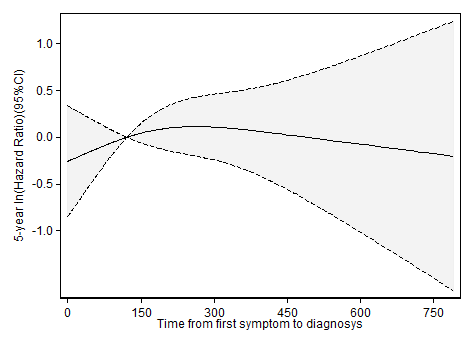
**

**Supplementary figure 2.-Cubic spline regression analysis of the relationship of time from symptom onset to diagnosis with 5-year CRC-specific survival in patients with colon cancer.**

**
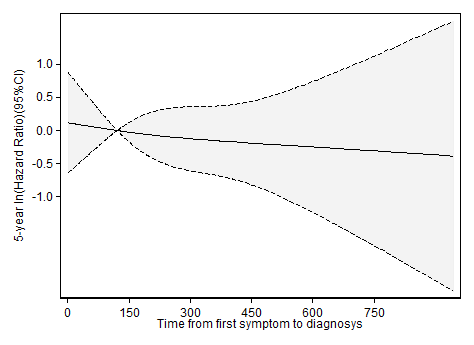
**

**Supplementary figure 3.-Cubic spline regression analysis of the relationship of time from symptom onset to diagnosis with 5-year CRC-specific survival in patients with rectal cancer.**
