# Supplementary figures and images for: Relationship between time from symptom’s onset to diagnosis and prognosis in patients with symptomatic colorectal cancer
Source: BMC Cancer. 2022 Aug 22;22:910. doi: 10.1186/s12885-022-09990-7 (PMC9394014; doi:10.1186/s12885-022-09990-7)

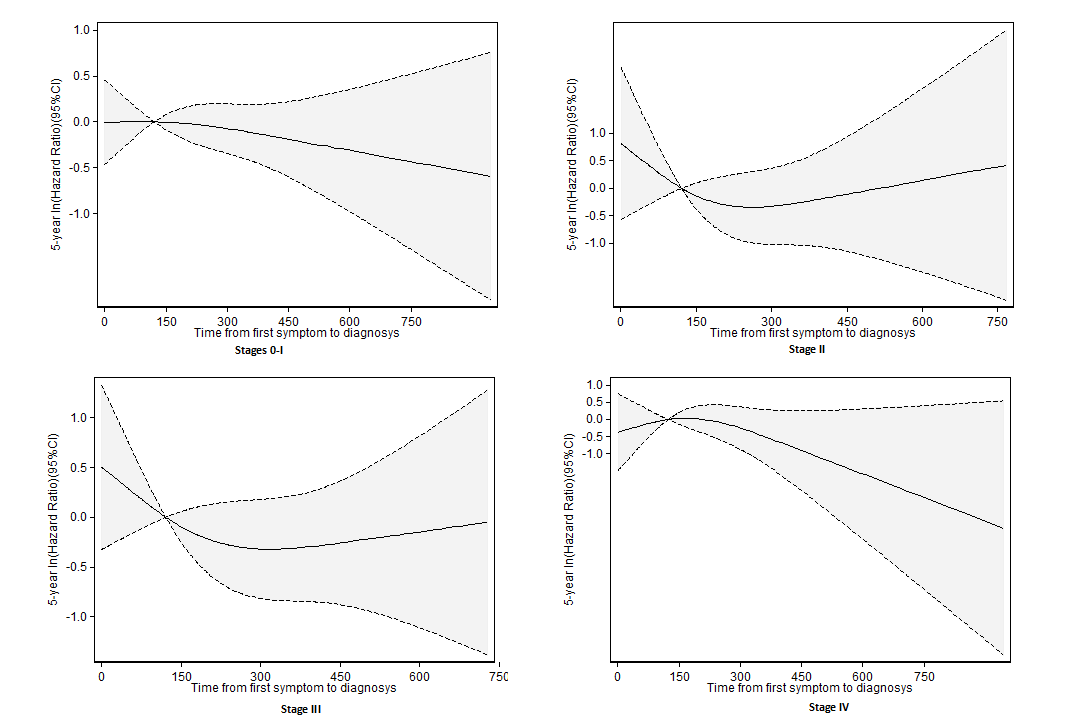

Supplement: Supplementary file 3 — Additional file 3. Supplementary fig 4 [file 12885_2022_9990_MOESM3_ESM.docx]
